# M2 macrophages mediate sorafenib resistance by secreting HGF in a feed-forward manner in hepatocellular carcinoma

**DOI:** 10.1038/s41416-019-0482-x

**Published:** 2019-05-27

**Authors:** Ningning Dong, Xiangyi Shi, Suihai Wang, Yanjun Gao, Zhenzhan Kuang, Qian Xie, Yonglong Li, Huan Deng, Yingsong Wu, Ming Li, Ji-Liang Li

**Affiliations:** 1 Institute of Antibody Engineering, School of Laboratory Medicine and Biotechnology, Southern Medical University, 510515 Guangzhou, China; 2 Wenzhou Medical University School of Biomedical Engineering and Eye Hospital, Wenzhou Institute of Biomaterials and Engineering, 325035 Wenzhou, China; 3 Institute of Translational and Stratified Medicine, University of Plymouth Faculty of Medicine and Dentistry, Plymouth, PL6 8BU UK

**Keywords:** Hepatocellular carcinoma, Cancer therapeutic resistance

## Abstract

**Background:**

Sorafenib is the only approved first line systemic therapy for advanced hepatocellular carcinoma (HCC) in the last decade. Tumour resistance to sorafenib has been of major obstacles to improve HCC patient survival.

**Methods:**

We polarised THP-1 cells to M1 and M2 macrophages, performed various in vitro assays and developed sorafenib-resistant xenograft models to investigate the role of tumour-associated macrophages (TAM)-secreted molecules in HCC resistance to the targeted therapy.

**Results:**

We demonstrated M2, but not M1, macrophages not only promote proliferation, colony formation and migration of hepatoma cells but also significantly confer tumour resistance to sorafenib via sustaining tumour growth and metastasis by secreting hepatocyte growth factor (HGF). HGF activates HGF/c-Met, ERK1/2/MAPK and PI3K/AKT pathways in tumour cells. Tumour-associated M2 macrophages were accumulated in sorafenib-resistance tumours more than in sorafenib-sensitive tumours in vivo and produced abundant HGF. HGF chemoattracts more macrophages migrated from surrounding area, regulates the distribution of M2 macrophages and increases hepatoma resistance to sorafenib in a feed-forward manner.

**Conclusions:**

Our results provide new insights into the mechanisms of sorafenib resistance in HCC and rationale for developing new trials by combining sorafenib with a potent HGF inhibitor such as cabozantinib to improve the first line systemic therapeutic efficacy.

## Background

Hepatocellular carcinoma (HCC) is the sixth most commonly diagnosed cancer and the fourth leading cause of cancer-related death worldwide,^1^ characterised by rapid progression with high post-operation recurrence and high metastasis.^2^ Currently, standardised treatments of HCC patients include surgical resection, liver transplantation, transcatheter arterial chemoembolization, local radiofrequency ablation, and systemic targeted therapy with sorafenib or lenvatinib in the first-line^3–5^ and regorafenib^6,7^ or nivolumab in the second-line setting after progression on sorafenib.^8,9^ Although early-stage of or localised HCC are curable by surgical resection, liver transplantation or local ablation, 80% of HCC patients are diagnosed at advanced disease stages when only systemic therapy with sorafenib followed by regorafenib or nivolumab shows to improve patient survival.^2^

Sorafenib, is a small-molecule inhibitor of up to 40 kinases, potently inhibiting proangiogenic receptor tyrosine kinases including VEGFR-1/2/3, PDGFR-β, and FGFR1, and other kinases involved in tumorigenesis (Raf-1, wild type B-Raf, mutant B-Raf, c-Kit, Flt-3, and RET).^10,11^ Preclinical studies have demonstrated sorafenib effectively inhibited tumour growth of various cancer types.^10^ In 2008, the SHARP phase III trial showed that sorafenib substantially increased median survival in patients with advanced stage of HCC from 7.9 to 10.7 months.^5^ The beneficial effect of sorafenib was validated in another independent Sorafenib-AP phase III trial that showed an extension of median survival from 4.2 to 6.5 months.^3^ As a result, sorafenib has become the standard of care for treatment of advanced HCC since 2007. However, due to intra-nodule and inter-nodule tumour heterogeneity and heterogeneity in tumour evolution,^12^ the response rate to sorafenib is very low and the effective duration is short in clinical trials,^3,5,13^ suggesting intrinsic primary and acquired secondary resistance. Indeed, tumour resistance to sorafenib has become a major obstacle to the effectiveness of systemic therapy against HCC since then. Thus, understanding of the resistance mechanisms and identification of molecular markers to stratify the patients for sorafenib therapy will improve the clinical benefits by developing new therapeutic approaches or rational drug combinations.^14^

Collective evidence shows that most studies on sorafenib resistance in HCC have been focused on tumour cells. Various mechanisms are involved in hepatoma resistance to sorafenib, including epithelial-mesenchymal transition (EMT) of tumour cells,^15^ cancer stem cells (CSC) or tumour-initiating cells,^16,17^ activation of numerous growth factor pathways such as AR/EGFR pathway^18^ and PI3K/AKT pathway,^19,20^ c-Jun activation,^21^ hypoxia,^22^ cancer cell metabolism,^23^ and autophagy,^24^ among others.^21^ However, growing evidence has also uncovered the importance of stroma cells in tumour microenvironment (TME) in HCC progression^25^ and response to sorafenib by cross-talking with tumour cells.^26^ These may include tumour-associated endothelia,^22^ tumour-associated neutrophils,^27^ cancer-associated fibroblasts,^28,29^ tumour-infiltrated lymphocytes such as NK cells^30^ and myeloid cells,^28^ and tumour-associated macrophages (TAM).^31–33^

We are interested in hepatocarcinogenesis and its potential translation for development of either novel targeted therapies or predictive markers for therapeutic efficacy and/or patient prognosis.^34^ In this paper, we report the role of M2-type of TAMs in hepatoma resistance to sorafenib by secreting hepatocyte growth factor (HGF). HGF activates HGF/c-Met, MAPK/ERK1/2, and PI3K/AKT pathways in tumour cells, further recruits M2 TAMs, and thus sustains hepatoma growth and metastasis in a feed-forward manner.

## Methods

### Cell lines and culture

Human acute monocytic leukaemia cell line THP-1 and hepatoma cancer cell lines (SMMC-7721, Hep3B, and Sk-hep1) were purchased from and authenticated by the Typical Culture Preservation Commission Cell Bank, Chinese Academy of Sciences (Shanghai, China). THP-1 cells were maintained in RPMI 1640 medium (Gibco BRL, New York, USA) while all hepatoma cells were cultured in DMEM (Gibco BRL, New York, USA) supplemented with 10% foetal bovine serum (FBS) (Gibco BRL, New York, USA) (complete medium) at 37 °C with 5% CO_2_.

### Polarisation of M1-like and M2-like macrophages

Production and polarisation of THP-1-derived macrophages were conducted as described previously^35,36^ with some modifications. Briefly, 1 × 10^7^ THP-1 cells in 10-cm dish were incubated in 10 ml complete medium containing 200 ng/ml PMA (Sigma) for 24 h and cultured in 10 ml fresh complete medium for another 24 h to produce THP-1 macrophages (M0). For M1 polarisation, the macrophages were then cultured in 10 ml fresh complete medium with 100 ng/ml LPS (Sigma) and 20 ng/ml IFN-γ (Sigma) for another 24 h; whereas for M2 polarisation the macrophages were cultured 10 ml fresh complete medium with 20 ng/ml IL-4 (Peprotech) and 20 ng/ml IL-13 (Peprotech) for 72 h. After polarisation, 10^6^ M1-like and M2-like macrophages were cultured in 10 ml serum-free RPMI 1640 medium for 48 h, respectively. The conditioned media (CM) were then harvested by centrifugation at 1300 rpm for 5 min and the supernatant was aliquoted and stored at −80 °C for further use.

### Flow cytometry analysis

After the polarisation, THP-1-derived macrophages were harvested and washed in PBS. 1 × 10^6^ cells were then stained using anti-CD11b-PE-Cy7, CD209-FITC, CD163-PE, CD115-PE-Cy7, CD204-FITC, CD206-APC (BD Pharmingen, USA) for 30 min at 4 °C. 1 × 10^6^ cells isolated from fresh xenograft tissues were stained using FITC-anti-mouse F4/80 (Bio-Rad), FITC-anti-mouse CD206 (Biolegend). An isotype-matched IgG was used as negative control. Results were analysed by flow cytometry (FACSCalibur, BD).

### RNA extraction and real-time quantitative PCR (RT-qPCR)

Total RNAs were extracted using Trizol reagent (Invitrogen) and converted into cDNA using the PrimeScript™ RT Master Mix (Takara). SYBR^®^ Premix Ex Taq™ (Takara) was then used for RT-qPCR with the Applied Biosystems 7500 software. GAPDH was used as an internal control. Relative gene expression at the mRNA level was calculated using 2^−ΔCT^ (ΔCT = Ct^target gene^ − CT^GAPDH^). Primer sequences for various genes are: HLA-DR forward (5′-ATCATGACAAAGCGCTCCAACTAT-3′), reverse (5′-GATGCCCACCAGACCCACAG-3′); MRC1 forward (5′-GGCGGTGACCTCACAAGTAT-3′), reverse (5′-TTTTCATGGCTTGGTTCTCC-3′); HGF forward (5′-CGAGGCCATGGTGCTATACT-3′), reverse (5′-GCATTCAGTTGTTTCCAAAGG-3′); TNF-α forward (5′-GGCTCCAGGCGGTGCTTG-3′), reverse (5′-CAGATAGATGGGCTCATACCA-3′); CXCL-9 forward (5′-GGACTATCCACCTACAATCCTTG-3′), reverse (5′- TTTTAATCAGTTCCTTCACATCTGC-3′; IL-10 forward (5′-GACTTTAAGGGTTACCTGGGTTG-3′), reverse (5′-TCACATGCGCCTTGATGTCTG-3′); IGF-1 forward (5′-GCTCTTCAGTTCGTGTGTGGA-3′), reverse (5′-GCCTCCTTAGATCACAGCTCC-3′);

VEGF forward (5′-CCTTGCTGCTCTACCTCCAC-3′), reverse (5′-GCAGTAGCTGCGCTGATAGA-3′); TGF-β forward (5′-CCCAGCATCTGCAAAGCTC-3′), reverse (5′-GTCAATGTACAGCTGCCGCA-3′); IL-12 forward (5′-CATCAGGGACATCATCAA-3′), reverse (5′-GTCAGGGAGAAGTAGGAA-3′); and GAPDH forward (5′-CCACCCATGGCAAATTCC-3′), reverse (5′-TGGGATTTCCATTGATGACAA-3′).

### Enzyme-linked immunosorbent assays (ELISA)

HGF in CM was measured by Human HGF ELISA Kit (Neobioscience, USA) on the Bio-Rad microplate reader (USA) following the manufacturer’s instructions.

### Cell proliferation assays

Total 1 × 10^4^ tumour cells (SMMC-7721, Hep3B or Sk-hep1) per well were in triplicate seeded in 24-well plates for 24 h and then incubated with the CM, 3 μM sorafenib (Sigma) (10 mM stock solution prepared with DMSO), 50 ng/ml recombinant human HGF (R&D), or 200 ng/ml anti-HGF neutralising antibody (Abcam) for another 48 h. Twenty microlitre Cell Counting Kit-8 (CCK-8; Sigma, USA) solution was then added to each well. After incubated at 37 °C for 4 h, the absorbance at 450 nm was measured on the Bio-Rad microplate reader. The experiment was repeated at least three times.

### Colony formation assays

Colony formation assays were performed with 6-well plates (500 or 1000 cells/well) as described previously.^34^ Briefly, an equal amount of different treatment cells was plated in 6-well plates triplicate. After incubated at 37 °C with 5% CO_2_ for 15–20 days, during which the supernatant in each well was replaced with fresh complete medium every 2–3 days, cell colonies formed were stained with 0.5% crystal violet following the fixation by methanol for counting. The experiment was repeated at least three times.

### Boyden chamber migration assays

To determine HCC cell migration affected by M1-like or M2-like THP-1-derived macrophages, tumour cells were seeded in the top of insert well in CM or serum-free RPMI 1640 medium (Corning), while medium containing 10% FBS was placed in the lower chamber. After incubation at 37 °C with 5% CO_2_, tumour cells migrated through the pores of insert well toward the chemoattractant below were stained in 0.5% crystal violet solution for 10 min and washed by running water for 10 min. Images were captured under microscope and the number of migrated cells were counted. The experiment was repeated at least three times.

### Western blotting

Western blotting was performed as described previously.^34^ Primary antibodies include rabbit anti-MET, pMET, ERK1/2, pERK1/2 (Thr202/Tyr204), AKT, and pAKT (Cell Signalling, USA). Primary rabbit anti-GAPDH antibody was used as a protein loading control. All assays were repeated at least three times.

### Xenograft models

Female BALB/c nude mice aged 4–6 weeks old were purchased from the Experimental Animal Centre at Southern Medical University and maintained under standard pathogen-free conditions. All experimental procedures were approved by the Ethical Committee of Southern Medical University and animal welfares were closely monitored in accordance with the Guide for the Care and Use of Laboratory Animals of the National Institutes of Health. Tumour xenografts were essentially conducted as described previously.^34^ Each group contained 5–7 mice. SMMC-7721 tumour cells (10^6^ in 100 μl) were subcutaneously implanted into each mouse. Tumour volume was calculated with the formula of [length × width^2^ × (*π*/6)]. When tumours reached 100 mm^3^ volume, mice were treated with sorafenib daily (30 mg/kg) via oral gavage.^37^ Mice were killed, and tumour cells were isolated from both sorafenib sensitive and resistant tumours as described in isolation of tumour tissue cells. Tumour cells isolated from the first round of xenograft tissues were cultured for ~48-h in vitro. Equal amount of tumour cells adhered on culture dishes from two mice (having similar tumour sizes) of either DMSO group or sorafenib group were then mixed (50:50% ratio) in each group. The mixed tumour cells were then for the second round of xenograft growth by subcutaneous injection of nude mice as potential sorafenib-sensitive (SS) and sorafenib-resistant (SR) tumours respectively. Sorafenib treatments were started when the second round of tumours reached 100 mm^3^. Mice were killed when tumour reached to 800 mm^3^. Tumours were then collected for either tissue CD31 staining or cell isolation for flow cytometry analysis of F4/80^+^ and CD206^+^ expressions.

### Isolation of tumour tissue cells

Fresh xenograft tumours were soaked in PBS and chopped to 1 mm^3^ size with surgical blade. After washed with PBS, the slices were digested with collagenase (type IV, 1.5 mg/ml, Sigma) and hyaluronidase (type I, 0.25 mg/ml, Sigma) DMEM culture medium by rotating at 37 °C for 2 h. Digested cells were filtered through a sterilised cell strainer (70 µm) and collected by centrifugation at 800 rpm/min for 5 min. After washed in 25 ml PBS, the isolated cells were either cultured for ~48-h for the second round of xenograft growth or resuspended in 5 ml RBC lysis buffer for 10 min. After washed in PBS, the cells were then aliquoted in 10^6^ cells/100 µl culture medium for flow cytometry analysis of F4/80^+^ and CD206^+^ expressions.

### Statistics

Data were presented as mean ± standard error of the mean (SEM). The SPSS 20.0 software (SPSS Inc., USA) and Prism 6 software were used for data analysis. The analysis of variance (ANOVA) test was used to compare mean values among three or more groups whereas independent-sample two-sided Student’s *t*-test was used to compare two groups with normal distribution data. Statistical significance was indicated by an asterisk (*P < 0.05, **P < 0.01, ***P < 0.001, and ****P < 0.0001).

## Results

### Polarisation of THP-1 to M1-like and M2-like macrophages

**Fig. 1:**
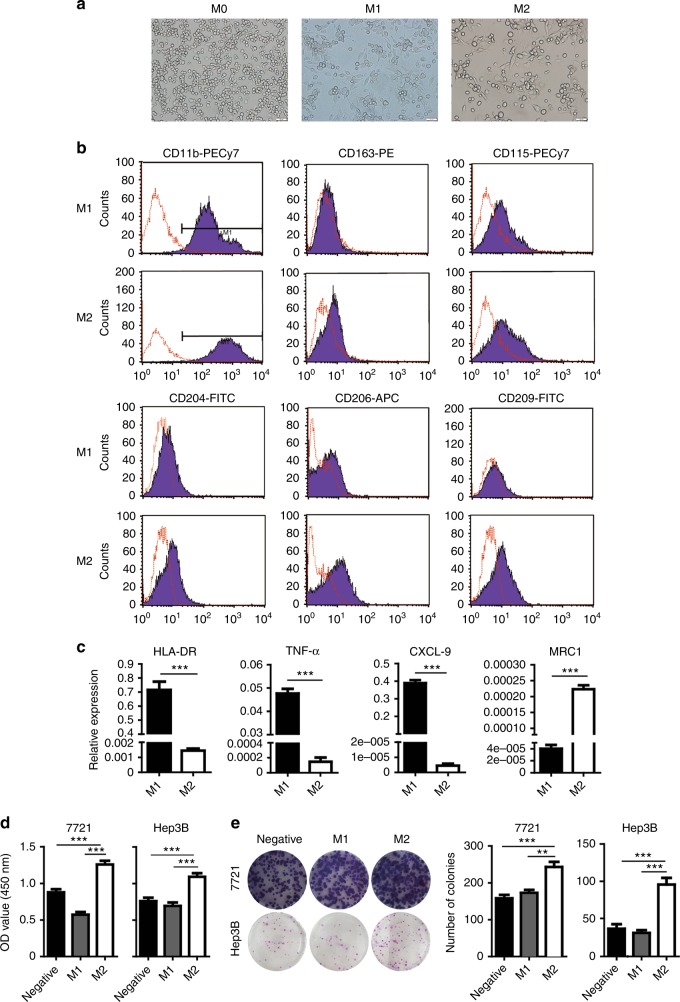
Polarisation of THP-1 to M1-like and M2-like macrophages. **a** Cell morphology of M0-like, M1-like, or M2-like macrophages. **b** Macrophage marker expressions of M1-like and M2-like macrophages were detected by flow cytometry assay using various specific antibody (black histograms) or isotype-matched antibody (red histogram). **c** Expressions of HLA-DR, TNF-α, CXCL-9 and MRC1 in M1 or M2 macrophages was determined by RT-qPCR. **d** Effects of M1-CM and M2-CM on the proliferation (at 48 h) of SMMC-7721 (left) and Hep3B cells (right), respectively. **e** Effects of M1-CM and M2-CM on the colony formation of SMMC-7721 and Hep3B cells respectively. *N* = 3, mean ± SEM, **P < 0.01, ***P < 0.001

To investigate the effect of macrophage-secreting proteins on innate resistance of HCC to sorafenib, we induced the differentiation of THP-1 cells with PMA to produce macrophages (M0) and then polarised the macrophages with LPS/IFN-γ and IL-4/IL-13, respectively.^35,36^ THP-1-derived M0 macrophages showed grape-like shapes while M1-like macrophages developed bipolar morphology in contrast to M2-like macrophages with clear bipolar extensions (Fig. 1a). Flow cytometry analysis demonstrated that CD11b was equally expressed in both M1-like and M2-like macrophages while the expression of CD163, CD115/CSF-1R, CD204/MSR1/SR-A, CD206/MRC1, or CD209/DC-SIGN was significantly higher in M2-like cells than that of M1-like cells (Fig. 1b). RT-qPCR analysis confirmed that M1 markers such as HLA-DR, CXCL9, and TNFα were significantly upregulated in M1-like macrophages polarised with LPS and IFN-γ whereas M2 marker, CD206/MRC1, was dramatically increased in THP-1-derived macrophages treated with IL-4 and IL-13 (Fig. 1c). Functional assays showed the abilities of proliferation and colony formation of either SMMC-7721 or Hep3B were significantly increased when incubated with CM from M2-like macrophages (M2-CM) compared to that of M1-like macrophages (M1-CM) (Fig. 1d, e). Together, the results suggested the polarised M1-like and M2-like THP-1 cells have intrinsic traits of tumour-associated M1 and M2 macrophages, respectively.

### M2 macrophages confer hepatoma resistance to sorafenib by secreting molecules

**Fig. 2:**
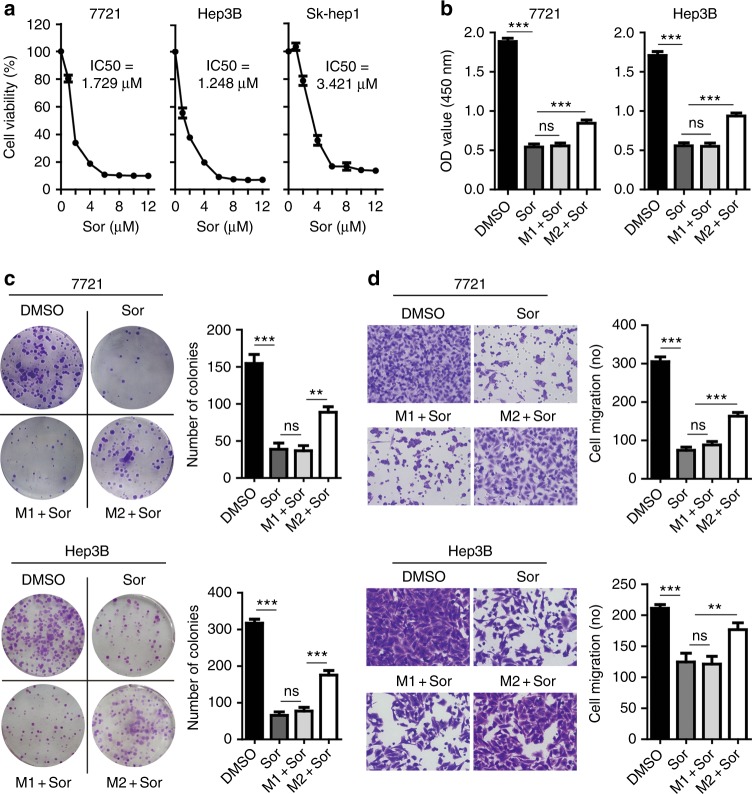
M2 macrophages confer hepatoma resistance to sorafenib by secreting molecules. **a** Sorafenib inhibited the growth of SMMC-7721, Hep3B, and Sk-hep1 cells cultured in serum-free medium for 48 h. IC50 of sorafenib against SMMC-7721, Hep3B, and Sk-hep1 is 1.729 μM, 1.248 μM, and 3.421 μM, respectively. **b** CCK-8 assays showing M2-CM but not M1-CM attenuated the ability of sorafenib (Sor) to inhibit the proliferation of SMMC-7721 or Hep3B cells. **c** Colony formation assays showing M2-CM but not M1-CM attenuated the ability of sorafenib to inhibit colony formation of SMMC-7721 or Hep3B cells. **d** Boyden chamber transwell assays showing M2-CM but not M1-CM attenuated the ability of sorafenib to inhibit cell migration of SMMC-7721 or Hep3B cells. *N* = 3, mean ± SEM, **P < 0.01, ***P < 0.001, NS, no significance

We first investigated the inhibitory efficacy of sorafenib against tumour cells growing in serum-free medium. We showed that IC50 of sorafenib against SMMC-7721, Hep3B, and Sk-hep1 was 1.729 μM, 1.248 μM, and 3.421 μM, respectively (Fig. 2a). We then treated tumour cells with sorafenib at 3 μM in the presence of CM harvested from the macrophages cultured at 48 h. The ability of cell proliferation (Fig. 2b) or colony formation (Fig. 2c) was significantly inhibited by sorafenib for both SMMC-7721 and Hep3B cells compared to the vehicle control (Sor versus DMSO, *P* < 0.001). However, tumour cells became highly resistant to sorafenib in the presence M2-CM (M2+Sor versus Sor, *P* < 0.001). In contrast, M1-CM showed no protection to tumour cells (M1+Sor versus Sor, *P* > 0.05). In addition, tumour migration was also significantly inhibited by sorafenib (Sor versus DMSO, *P* < 0.001) (Fig. 2d). Again, tumour migration was significantly increased by M2-CM (M2+Sor versus Sor, *P* < 0.001) but not by M1-CM (M1+Sor versus Sor, *P* > 0.05). Thus, the results suggested that M2 macrophages confer hepatoma resistance to sorafenib via sustaining tumour growth and metastasis by secreted soluble molecules.

### HGF mediates sorafenib resistance conferred by M2 macrophages

**Fig. 3:**
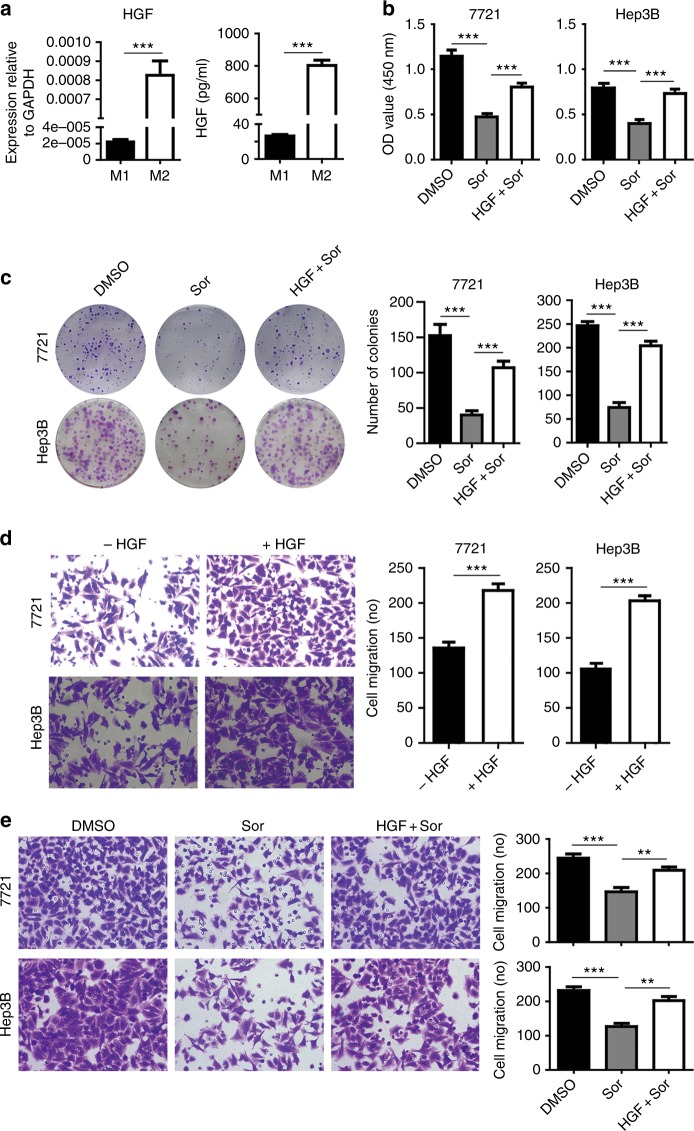
HGF in CM mediates sorafenib resistance conferred by M2 macrophages. **a** M2 macrophages expressed and secreted much higher HGF than that of M1 macrophages as determined by RT-qPCR (at mRNA level) and by ELISA assays (protein in CM) after macrophages were cultured in serum-free medium for 48 h. **b** Recombinant human HGF (50 ng/ml) reduced the inhibitory efficacy of sorafenib on the proliferation of SMMC-7721 or Hep3B cells as measured by CCK-8 assays. **c** Colony formation assays showing recombinant HGF impaired sorafenib efficacy on suppression of SMMC-7721 or Hep3B proliferation. **d** Transwell assays showing recombinant HGF significantly increased the migration of SMMC-7721 or Hep3B cells. **e** Recombinant HGF impaired the suppressive efficacy of sorafenib on the migration SMMC-7721 or Hep3B cells. *N* = 3, mean ± SEM, **P < 0.01, ***P < 0.001

To investigate which molecules secreted from M2 macrophages are responsible for the sorafenib resistance, we screened a variety of gene expressions differentially between M1 and M2 macrophages at mRNA levels by RT-qPCR (Fig. S1). Of them, HGF expression was greatly upregulated in M2 macrophages compared to that of M1 macrophages (Fig. 3a, left panel). ELISA revealed that the amount of HGF protein in CM secreted by M2 macrophages was 30-folds more than that of M1 macrophages (Fig. 3a, right panel). We then investigated if exogenous HGF confers HCC resistance to sorafenib by performing proliferation and colony formation assays through incubation of tumour cells with 50 ng/ml recombinant human HGF. Exogenous HGF significantly increased the growth and colony formation of tumour cells in the presence of sorafenib (Fig. 3b, c, HGF+Sor versus Sor, *P* < 0.001). Also, exogenous HGF remarkably increased HCC migration (Fig. 3d) and significantly abolished the inhibitory effect of sorafenib on the migration of SMMC-7721 and Hep3B cells (Fig. 3e).

**Fig. 4:**
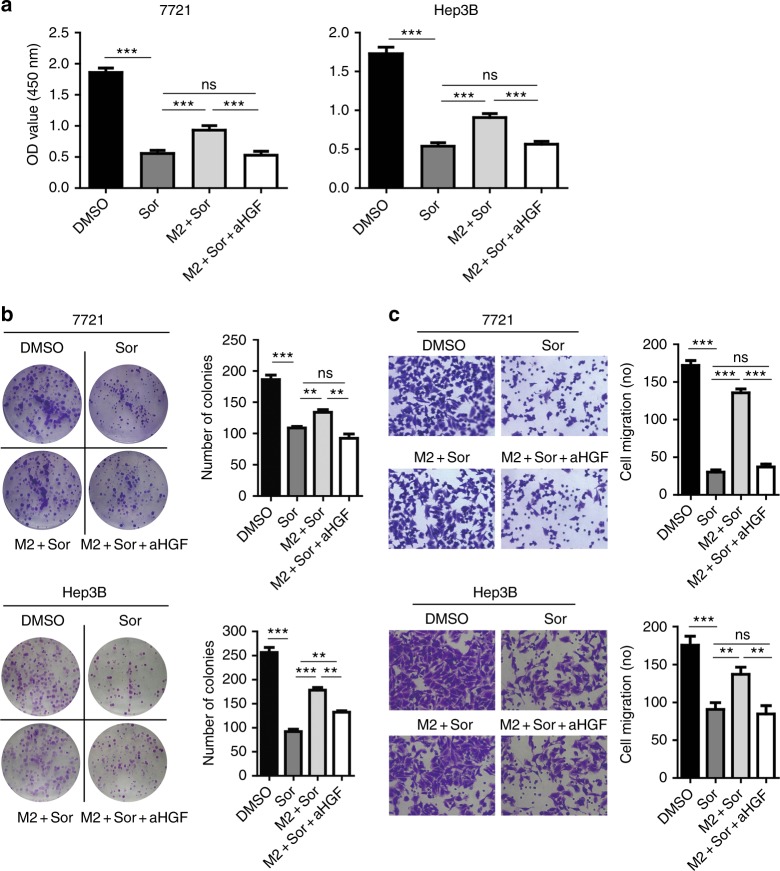
Anti-HGF antibody abolished the ability of M2-CM to promote the proliferation, colony formation and migration of hepatoma cells. **a** After incubation of SMMC-7721 and Hep3B cells with 3 μM sorafenib in the presence or absence of anti-HGF antibody (aHGF) (200 ng/ml) for 48 h, cell proliferation was examined by CCK-8 assays. **b** Anti-HGF antibody abolished the effect of M2-CM on sorafenib inhibition of the colony formation of SMMC-7721 and Hep3B cells. **c** Anti-HGF antibody abolished the effect of M2-CM on the sorafenib inhibition of the migration of SMMC-7721 and Hep3B cells. *N* = 3, mean ± SEM, **P < 0.01, ***P < 0.001, NS, no significance

To investigate if HGF produced by M2 macrophages directly confers HCC resistance to sorafenib, we added anti-HGF antibody (200 ng/ml) to neutralise the HGF activity in M2-CM. As shown in Fig. 4, M2-CM increased cell proliferation, colony formation and migration of both SMMC-7721 and Hep3B (M2+Sor versus Sor, all *P* < 0.01). However, addition of anti-HGF antibody abolished the proliferation (Fig. 4a), colony formation (Fig. 4b), and migration (Fig. 4c) of both SMMC-7721 and Hep3B cells promoted by M2-CM (M2+Sor+aHGF versus M2+Sor, all *P* < 0.01), suggesting a dominant role of HGF in sorafenib resistance conferred by M2-CM.

### M2 macrophages confer sorafenib resistance by activation of ERK/MAPK and PI3K/AKT pathways through HGF/c-Met signalling

**Fig. 5:**
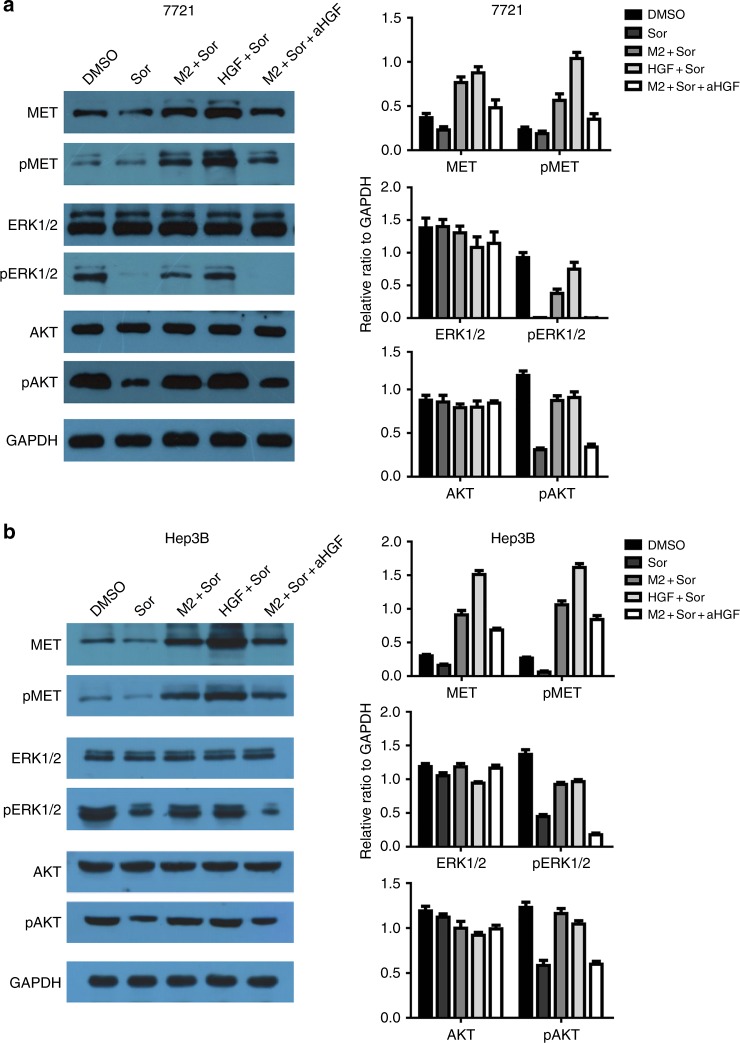
M2 macrophages confer sorafenib resistance by activation of ERK/MAPK and PI3K/AKT pathways through HGF/c-Met signalling. **a** Treatment with sorafenib, M2-CM, HGF or anti-HGF antibody (aHGF) alone or combination affects the expression and phosphorylation of c-Met, ERK and AKT kinases in SMMC-7721 cells as measured by Western blots. GAPDH is a protein loading control. **b** Treatment with sorafenib, M2-CM, HGF or anti-HGF antibody (aHGF) alone or combination affects the expression and phosphorylation of c-Met, ERK, and AKT kinases in Hep3B cells as measured by Western blots. GAPDH is a protein loading control

To explore the possible mechanisms by which M2 macrophages induce sorafenib resistance, we investigated the expression and phosphorylation of key components in HGF/c-Met, ERK/MAPK, and PI3K/AKT pathways in tumour cells of SMMC-7721 and Hep3B from different treatment groups. Western blot analysis showed that sorafenib treatment did not significantly affect the expression and phosphorylation of c-Met (Fig. 5a, b). However, incubation of tumour cells with either M2-CM or exogenous HGF significantly increased the expression and phosphorylation of c-Met. Neutralising HGF by addition of anti-HGF antibody eliminated the increment of c-Met expression and phosphorylation enhanced by M2-CM or recombinant HGF, suggesting a dominant role of HGF in M2-CM in the sorafenib resistance.

Sorafenib did not affect the expression of ERK1/2 but dramatically reduced the phosphorylation level of both pERK1/2, confirming the role of sorafenib in inhibition of ERK1/2/MAPK pathway by targeting RAF.^10^ Incubation of tumour cells with either M2-CM or exogenous HGF partially abolished the suppression of pERK1/2 phosphorylation by sorafenib. However, neutralising HGF in M2-CM by anti-HGF antibody sustained the function of sorafenib, supporting the role of HGF in M2-CM in induction of pERK1/2 phosphorylation. Similar results were obtained for the expression and phosphorylation of AKT, suggesting that the effect of M2-CM on ERK/MAPK and PI3K/AKT signalling is only partially mediated by HGF/c-Met signalling.

### Accumulation of tumour-associated macrophages in sorafenib-resistant tumour

**Fig. 6:**
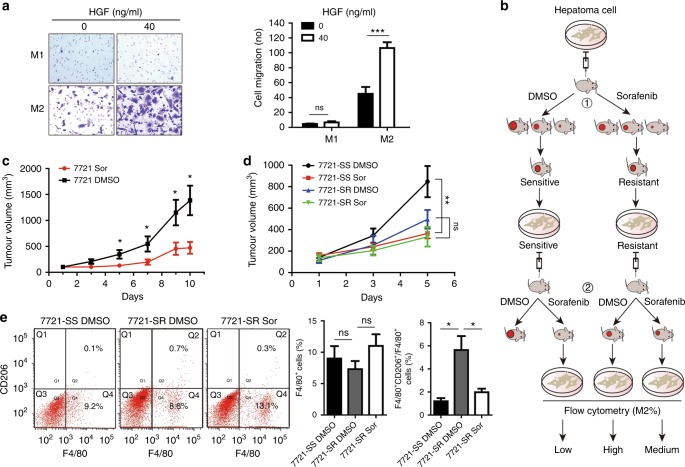
HGF chemoattracts the migration of tumour-associated M2 macrophages accumulated in sorafenib-resistant tumours. **a** HGF chemoattracts the migration of M2 macrophage but not M1 macrophage as determined by transwell migration assays. **b** Experimental procedures for development of sorafenib-resistant xenograft models by passage of HCC cells in nude mice, inducible treatment of mice with sorafenib, and implantation of mouse-passaged tumour cells in mice again. **c** Daily sorafenib treatment inhibited hepatoma growth in immunocompromised mice for the first round of xenograft. *N* = 5–6, Mean ± SEM, *P < 0.05. Tumour growth in individual mouse was shown in Supplementary Fig. 2a. **d** Daily sorafenib treatment of the mice greatly inhibited the tumour growth on sorafenib-sensitive (SS) tumour models (SS-Sor versus SS-DMSO, *P* < 0.01) but marginally affected the tumour growth on sorafenib-resistant (SR) tumour models (SR-Sor versus SR-DMSO, *P* > 0.05). *N* = 5–7, mean ± SEM, **P < 0.01, NS, no significance. **e** Flow cytometry assays showing SR-DMSO tumour contains more F4/80^+^ and CD206^+^ double positive M2 macrophages than that of SR-Sor tumour [SR-DMSO (*N* = 3) versus SR-Sor (*N* = 4), *P* < 0.05] or SS-DMSO tumour [SR-DMSO (*N* = 3) versus SS-DMSO (*N* = 3), *P* < 0.05)]. Mean ± SEM, *P < 0.05, NS, no significance

To investigate if HGF regulates recruitment of TAMs in tumour microenvironment, we initially performed Boyden chamber migration assays. M2 macrophages showed strong intrinsic migratory ability. Exogenous HGF significantly chemoattracted the migration of M2 macrophages but not M1 macrophages (Fig. 6a), suggesting that HGF secreted by M2-like TAMs may recruit more macrophages in tumour tissues, regulate the distribution of M2 macrophages and increase tumour resistance to sorafenib in a feed-forward manner.

We then developed SS and SR tumour models by passaging SMMC-7721 cells in immunocompromised mice for two rounds (Fig. 6b).^16^ Tumour cells isolated from the first round of xenografted tumours in DMSO control group (D1 and D5) and in sorafenib treatment group (S4 and S5) (Figs. 6c, S2) were further implanted in mice to generate SS tumour and SR tumour respectively. Daily treatment with sorafenib greatly suppressed the growth of SS tumour and reduced the tumour size by day 5 (SS-Sor versus SS-DMSO, *P* < 0.01); however, the treatment only marginally inhibited the growth of SR tumour and reduced the tumour size by day 5 (SR-Sor versus SR-DMSO, *P* > 0.05) (Fig. 6d), confirming the sorafenib resistance. Vascular examination by CD31 staining on xenograft sections did not show significant difference of the endothelial density between SR-DMSO tumour and SS-DMSO tumour; however, more larger vessels were visualised in SR-DMSO tumour than in SS-DMSO tumour and these larger vessels appeared to be less sensitive to sorafenib treatment (Fig. S3). We then investigated the expression of macrophage (F4/80) and M2 macrophage (CD206) markers on cells isolated from xenograft tissues by flow cytometry (Fig. 6e). F4/80^+^ cells isolated from SR-Sor tumour were slightly higher than that of SR-DMSO tumour or SS-DMSO tumour but no statistical difference (SR-Sor versus SR-DMSO or SR-Sor versus SS-DMSO, *P* > 0.05); however, F4/80^+^/CD206^+^ double positive cells isolated from SR-DMSO tumour were significantly higher than that of SR-Sor tumour or SS-DMSO tumour (either SR-DMSO versus SR-Sor or SR-DMSO versus SS-DMSO, *P* < 0.05), highlighting the role of M2-like TAMs in sorafenib resistance.

## Discussion

Macrophages, a major component in TME,^38^ are mainly recruited from the blood circulation monocytes and resided in tumour tissues (TAM) by cytokine CSF-1, chemokines CCL2, CCL9, CCL17, CCL18, and periostin. TAMs have the potential to elicit tumour-destructive reactions (antitumour activity) or drive tumour initiation, angiogenesis, metastasis and suppression of T cell immunity (pro-tumour activity).^39^ In HCC, the role of TAMs in clinical outcomes remains controversial^40–43^ which may due to the high plasticity and heterogeneity of TAMs. In fact, macrophages are able to differentiate to classically (M1, to mirror T_H_1) and alternatively (M2, to mirror T_H_2) activated macrophages under certain circumstances at the extremes of continuum of functional spectrum. These populations are often distinguished by inducing stimuli (e.g., CSF-1, LPS, and IFN-γ for M1; GM-CSF, IL-4 and IL-13 for M2), secretion profiles (e.g., IL-12^high^, IL-6^high^, TNFα^high^, and CXCL9^high^ for M1; IL-10^high^ and TGF-β1^high^ for M2), protein markers (e.g., HLA-DR and NOS2 for M1; CD163, CD204/MSR1/SR-A, CD206/MRC1, CD115/CSF-1R, and Arg1 for M2), and functional characteristics (e.g., antitumour or pro-tumour activity).^44–46^ CD11b and CD68 are among the markers commonly used to identify pan-monocyte/macrophages.

It has been demonstrated in HCC that M2 macrophages can improve EMT tumour metastasis through production IL-1β and HGF^47^ while M1 macrophages inhibits tumour metastasis by distinct integrin–Rho GTPase–Hippo pathways.^26^ Interestingly, sorafenib was recently reported to induce EMT and promote invasiveness and metastasis of HCC cells by downregulation of HTATIP2 expression via JAK-STAT3 signalling in orthotopic mouse models.^48^

We investigated the role of TAMs in sorafenib resistance in HCC. To do this, we first used the human monocytic cell line THP-1 to establish M1 macrophage and M2 macrophage models by differentiating the cells with PMA and consequently polarised the differentiated macrophages with LPS plus IFN-γ or IL-4 plus IL-13 respectively.^35,36^ Identifications of morphological phenotypes, gene expression genotypes and macrophage marker expressions demonstrated that the THP-1-derived M1 and M2 macrophages resemble to tumour-associated M1 and M2 macrophages respectively in multiple aspects. Co-culture of HCC cells (SMMC 7721, Hep3B, and Sk-Hep1) with THP-1 CM showed M1 macrophages did not affect the proliferation and colony formation of tumour cells while M2 macrophages significantly promoted the proliferation and colony formation. Sorafenib potently inhibited the growth, colony formation, and migration of hepatoma cells. Intriguingly, M2-CM significantly attenuated the abilities of sorafenib to inhibit the growth, colony formation and migration of hepatoma cells while M1-CM did not have such effects, suggesting that M2 but not M1 macrophages contribute to sorafenib resistance in HCC by secreting soluble factor(s).

It is known that TAMs produce a variety of factors,^38,39^ some of which such as CCL22 and IGF-1 may contribute to sorafenib resistance.^28,29,31,49^ By screened gene expressions differentially between M1 and M2 macrophages, we found M2 macrophages express HGF abundantly and secret approximately 30-folds of HGF more than M1 macrophages did. Substitute of M2-CM with recombinant human HGF mitigated the capabilities of sorafenib to inhibit the growth, colony formation and migration of hepatoma cells. Addition of anti-HGF to M2-CM abolished the activities of M2-CM on sorafenib, suggesting that HGF in M2-CM play the predominant role in hepatoma resistance to sorafenib conferred by M2 macrophages via sustaining tumour growth and metastasis. Apart from tumour-associated M2 macrophages, cancer-associated fibroblasts and hepatoma cells^50,51^ also produce a significant amount of HGF that may contribute to the acquisition of sorafenib resistance. To further explore underlying mechanisms, we assessed the expression and phosphorylation of key proteins in HGF/c-Met, ERK/MAPK and PI3K/AKT pathways in hepatoma cells. We found that sorafenib did not significantly affect protein expressions of c-Met, ERK1/2 and AKT as well as the phosphorylation of pMet, but significantly decreased the phosphorylation of pERK/1/2 and pAKT, confirming the role of sorafenib in inhibition of ERK1/2/MAPK pathway by targeting RAF.^10,11^ Combination of M2-CM or recombinant HGF with sorafenib significantly increased the expression of c-Met and the phosphorylations of pMet, pERK1/2, and pAKT; adding anti-HGF antibody to the combination of M2-CM and sorafenib had the same effects as sorafenib alone. Taken together, the results suggest that (a) sorafenib itself does not affect HGF/c-Met signalling but inhibits ERK1/2/MAPK signalling by targeting RAF, and PI3K/AKT signalling by the ERK1/2/MAPK pathway or other mechanism(s); (b) HGF secreted by M2 macrophages activates HGF/c-Met signalling and the downstream ERK1/2/MAPK and PI3K/AKT pathways.^52,53^ Indeed, PI3K has been shown to be involved in sorafenib resistance in HCC.^19,20^

In this study, we show HGF promotes the migration of both hepatoma cells and M2 macrophages, suggesting HGF secreted by tumour-associated M2 macrophages may recruit more macrophages into tumours, regulate the distribution of M2 macrophages in tumour tissues, and thus increase tumour resistance to sorafenib in a feed-forward manner. We further developed xenograft sorafenib-resistant and sorafenib-sensitive models by passage of HCC cells in nude mice, inducible treatment of mice with sorafenib, and implantation of mouse-passaged tumour cells in mice again.^16^ We found F4/80^+^/CD206^+^ double positive M2 macrophages are significantly higher in SR tumour than that of SS tumour. Short-term sorafenib treatment decreased the amount of F4/80^+^/CD206^+^ M2 macrophages (see Fig. 6e) but long-term sorafenib exposure increased HGF synthesis and secretion, along with increased levels of c-Met and pMet.^50,51^ Since sorafenib is also an antiangiogenic agent, the anti-tumour effect might be associated with the antiangiogenic activity,^54^ off-tumour adverse effects^55^ and even tumour metastasis^56^ under certain circumstances.^57^ We examined tumour vasculature in SR and SS tumours by CD31 staining. No significant difference for the endothelial density was observed between SR tumour and SS tumour but more larger vessels existed in SR tumour than in SS tumour, suggesting that such larger vessels may also contribute to sorafenib resistance.^37^

In HCC clinical samples, it is noted that CD206^+^ M2 macrophages were highly accumulated in peritumour area,^49^ a fraction of which highly expressed c-Met.^58^ Increased c-Met expression was independently associated with poor survival in multivariate analysis.^59^ Importantly, a recent biomarker analysis of samples from the pivotal phase III trial of sorafenib showed a trend toward improved survival in patient with a lower pre-treatment plasma HGF concentration,^60^ highlighting the clinical importance of tumour-associated M2 macrophages and HGF in tumour response to sorafenib. Cabozantinib, another multiple kinase inhibitor with most potent inhibition of c-Met, was recently shown to improve median overall survival from 8.0 to 10.2 months as a second line agent in the CELESTIAL phase III trial of HCC.^61^ Taken all together, we postulated combination of sorafenib with cabozantinib will be likely to improve the first line systemic therapeutic efficacy.

In conclusion, we demonstrated that tumour-associated M2 macrophages but not M1 macrophages increase the growth and migration of hepatoma cells, confer hepatoma resistance to sorafenib treatment by secreting HGF. HGF from M2 macrophages and tumour cells activates HGF/c-Met, MAPK/ERK1/2, and PI3K/AKT pathways in tumour cells, recruits more macrophages from surrounding blood circulation, regulates the distribution of M2 macrophages in tumour tissues, and increases tumour resistance to sorafenib in a feed-forward manner (Fig. S4). Our results provide new insights into sorafenib resistance in HCC and advocate the development of new trials for the first line systemic therapy by combination of sorafenib with a potent HGF inhibitor such as cabozantinib to improve the first line systemic therapeutic efficacy.

## Supplementary information

Supplementary Files

## Data Availability

All data and materials generated during and/or analysed during the current study are available from the corresponding author on reasonable request.
