# Supplementary material for: M2 macrophages mediate sorafenib resistance by secreting HGF in a feed-forward manner in hepatocellular carcinoma
Source: Br J Cancer. 2019 May 27;121(1):22–33. doi: 10.1038/s41416-019-0482-x (PMC6738111; doi:10.1038/s41416-019-0482-x)
Supplement: Supplementary file 1 — Supplementary Files [file 41416_2019_482_MOESM1_ESM.docx]

**Supplementary Figures and Legend**

**
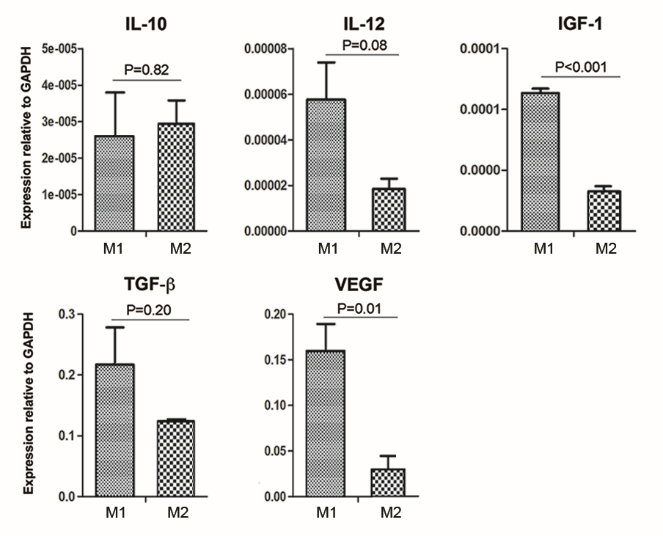
**

**Figure S1. Gene expressions differentially between M1-like and M2-like macrophages derived from THP-1 as detected by RT-qPCR.**

**
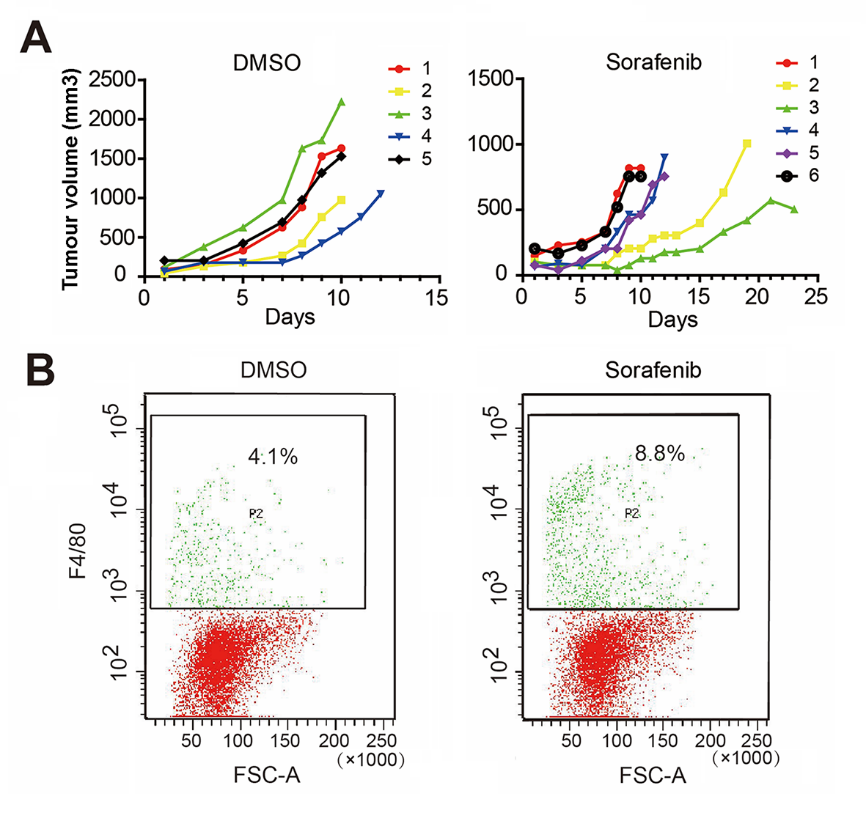
**

**Figure S2. Development of sorafenib-resistant xenograft models.**

**(A)** Tumour growth in individual mouse described in Figure 6BC. There were 5 mice in DMSO control group but 6 mice in sorafenib treatment group. Tumour cells were successfully isolated from D1, D4 and D5 xenograft tissues in DMSO group as well as from S1, S4 and S5 in sorafenib group respectively. After up to 48-hr cultivation *in vitro*, an equal amount of adhered tumour cells from D1 and D5 (having similar tumour sizes) in DMSO group or from S4 and S5 (having similar tumour sizes) in sorafenib group were mixed in 50:50 ratio, respectively. The resultant tumour cells from D1/D5 or from S4/S5 were for the second round of xenograft growth by injecting to nude mice subcutaneously as sorafenib-sensitive (SS) and sorafenib-resistant (SR) tumours respectively shown in Figure 6D.

**(B)** Flow cytometry assays showing tumour from sorafenib group appeared to contain more F4/80+ cells (8.8%) than that (4.1%) of tumour from DMSO group.


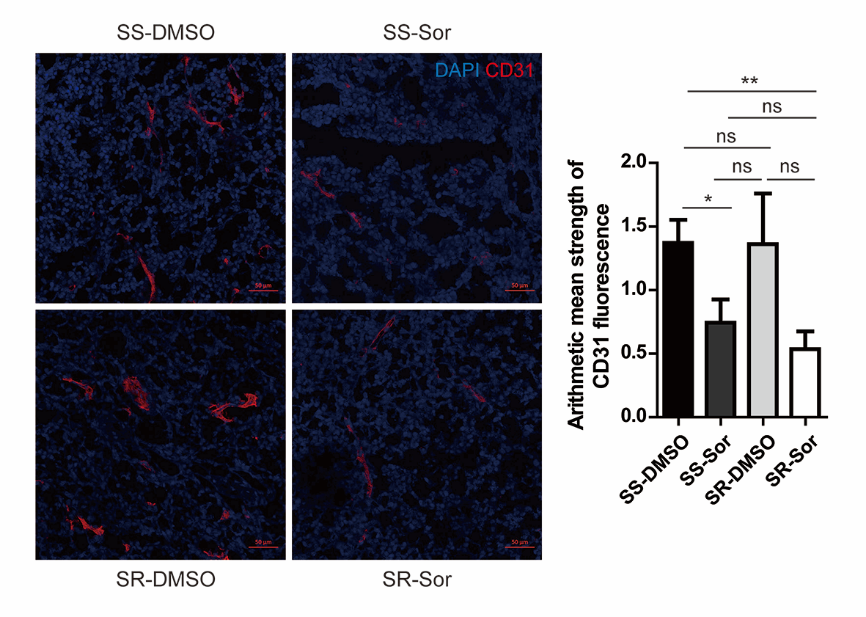


**Figure S3. Tumour vascular alternations in sorafenib-resistant and sorafenib-sensitive xenograft models as detected by tissue CD31 staining.** Mean ± SEM, N=5, *P<0.05, **P<0.01, NS, no significance.

**
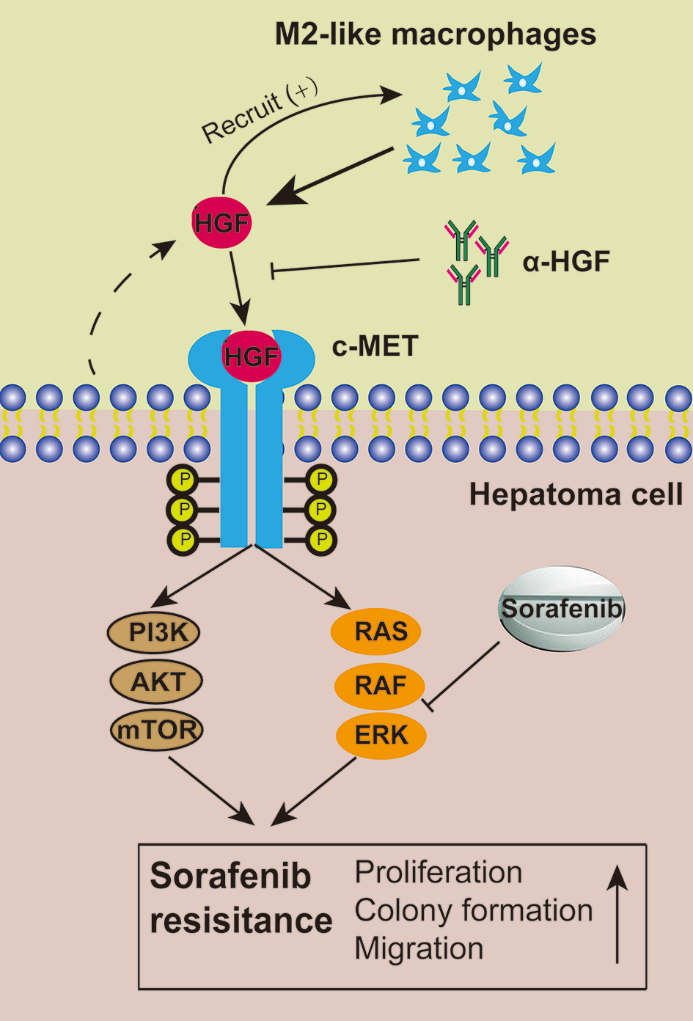
**

**Figure S4.** Summary of the working model. HGF secreted abundantly from tumour-associated M2 macrophages and less from tumour cells activates HGF/c-Met, MAPK/Erk1/2 and PI3K/AKT pathways, increases proliferation, colony formation and migration of tumour cells as well as tumour resistance to sorafenib. Abundant HGF also chemoattracts macrophages migrated from surrounding area (blood circulation monocytes), regulates the distribution of M2 macrophages in tumour tissues, and increases tumour resistance to sorafenib in a feed-forward manner.
